# Supplementary material for: A Likelihood Approach for Real-Time Calibration of Stochastic Compartmental Epidemic Models
Source: PLoS Comput Biol. 2017 Jan 17;13(1):e1005257. doi: 10.1371/journal.pcbi.1005257 (PMC5240920; doi:10.1371/journal.pcbi.1005257)
Supplement: S1 File — (TAR.GZ) [file pcbi.1005257.s014.tar.gz › HSPH_Online-SI-Revision/Additions/MSS11/n100-extreme-SEITR_noisy/n100-extreme-SEITR_noisy_NEW2_table0.pdf]

1	newly 3 weeks spec	6225.94	6598.74	{6487., 6712.}	5.9878	0.0252168	newly 3 weeks cumul	27 440.5	23 855.4	{23 658., 24 000.}	13.065	0.0608123	newly 5 weeks spec	2726.36	4185.27	{4110., 4270.}	53.5111	0.186088	newly 5 weeks cumul	34 535.3	33 342.1	{33 110., 33 529.}	3.45517	0.0152756
	newly 50 weeks cumul	41 541.4	47 013.5	{46 809., 47 182.}	13.1727	0.0537399																		
2	newly 3 weeks spec	7321.	6788.96	{6650., 6917.}	7.26731	0.0328077	newly 3 weeks cumul	36 564.	31 890.	{31 640., 32 112.}	12.7832	0.0594061	newly 5 weeks spec	2664.	2600.89	{2521., 2672.}	2.63326	0.0116471	newly 5 weeks cumul	43 633.	38 747.9	{38 527., 38 983.}	11.1959	0.0515711
	newly 58 weeks cumul	47 260.	42 676.8	{42 427., 42 882.}	9.69793	0.044306																		
3	newly 3 weeks spec	8747.	4330.97	{3485., 6388.}	50.4862	0.320627	newly 3 weeks cumul	40 258.	28 000.2	{24 869., 35 754.}	30.4482	0.163232	newly 5 weeks spec	3413.	945.67	{653., 1598.}	72.2921	0.589954	newly 5 weeks cumul	49 075.	30 996.7	{27 147., 40 586.}	36.8381	0.20643
	newly 60 weeks cumul	54 208.	31 795.8	{27 696., 42 078.}	41.3449	0.239201																		
4	newly 3 weeks spec	8496.	5422.56	{5127., 5438.}	36.1751	0.196981	newly 3 weeks cumul	36 998.	28 166.6	{27 365., 28 122.}	23.87	0.119089	newly 5 weeks spec	3304.	1664.78	{1484., 1674.}	49.6132	0.304534	newly 5 weeks cumul	45 863.	32 889.8	{31 817., 32 656.}	28.2869	0.145435
	newly 58 weeks cumul	50 745.	34 810.4	{33 543., 34 404.}	31.4013	0.165173																		
5	newly 3 weeks spec	5120.	5040.8	{4952., 5130.}	1.75195	0.00769772	newly 3 weeks cumul	31 803.	29 600.3	{29 387., 29 816.}	6.92617	0.0311781	newly 5 weeks spec	1318.	1400.63	{1349., 1456.}	6.37557	0.0266787	newly 5 weeks cumul	35 687.	33 670.2	{33 457., 33 869.}	5.65139	0.0252694
	newly 60 weeks cumul	37 045.	35 159.7	{34 921., 35 372.}	5.0893	0.0226901																		
6	newly 3 weeks spec	4778.	6303.6	{6212., 6402.}	31.9297	0.120312	newly 3 weeks cumul	31 868.	31 671.8	{31 479., 31 893.}	0.702272	0.0030638	newly 5 weeks spec	1057.	2217.86	{2133., 2289.}	109.826	0.32171	newly 5 weeks cumul	35 197.	37 665.1	{37 469., 37 907.}	7.01236	0.0294297
	newly 59 weeks cumul	35 989.	40 712.9	{40 502., 40 936.}	13.1259	0.0535586																		
7	newly 3 weeks spec	7357.	6423.7	{6317., 6540.}	12.6859	0.0589562	newly 3 weeks cumul	28 195.	23 998.5	{23 832., 24 194.}	14.8838	0.0699966	newly 5 weeks spec	3977.	3837.59	{3765., 3907.}	3.53256	0.0156633	newly 5 weeks cumul	37 670.	32 863.6	{32 642., 33 070.}	12.7594	0.0592861
	newly 51 weeks cumul	47 209.	44 174.1	{43 970., 44 377.}	6.42858	0.0288596																		
8	newly 3 weeks spec	7216.	5865.89	{5727., 5967.}	18.71	0.0900145	newly 3 weeks cumul	32 722.	29 204.4	{28 943., 29 422.}	10.7498	0.0493998	newly 5 weeks spec	3068.	2060.67	{1997., 2112.}	32.8334	0.172964	newly 5 weeks cumul	40 537.	34 771.6	{34 527., 35 020.}	14.2226	0.0666334
	newly 58 weeks cumul	46 063.	37 562.8	{37 299., 37 817.}	18.4535	0.0886																		
9	newly 3 weeks spec	5164.	5033.75	{4928., 5152.}	2.61367	0.0115477	newly 3 weeks cumul	32 212.	29 589.8	{29 395., 29 775.}	8.14048	0.0368809	newly 5 weeks spec	1330.	1394.42	{1340., 1448.}	4.98496	0.0209617	newly 5 weeks cumul	36 196.	33 660.3	{33 455., 33 833.}	7.00536	0.0315469
	newly 60 weeks cumul	37 584.	35 150.5	{34 956., 35 326.}	6.47491	0.0290757																		
10	newly 3 weeks spec	8694.	7385.11	{7259., 7510.}	15.0551	0.070907	newly 3 weeks cumul	33 638.	29 943.7	{29 662., 30 140.}	10.9827	0.0505487	newly 5 weeks spec	4707.	3485.57	{3396., 3567.}	25.9492	0.130551	newly 5 weeks cumul	44 803.	38 609.8	{38 300., 38 805.}	13.8231	0.0646276
	newly 56 weeks cumul	56 292.	45 181.5	{44 861., 45 449.}	19.7372	0.095501																		
11	newly 3 weeks spec	8565.	7860.54	{7739., 7967.}	8.22487	0.0373039	newly 3 weeks cumul	34 772.	31 035.4	{30 827., 31 267.}	10.7459	0.0493786	newly 5 weeks spec	4255.	3888.91	{3810., 3969.}	8.60376	0.0391279	newly 5 weeks cumul	45 069.	40 556.2	{40 346., 40 774.}	10.0131	0.0458252
	newly 55 weeks cumul	54 734.	48 415.3	{48 160., 48 655.}	11.5444	0.053278																		
12	newly 3 weeks spec	7136.	5545.31	{5451., 5668.}	22.2911	0.10958	newly 3 weeks cumul	31 568.	25 871.4	{25 669., 26 070.}	18.0456	0.086437	newly 5 weeks spec	3139.	2103.79	{2039., 2174.}	32.979	0.173912	newly 5 weeks cumul	39 318.	31 433.8	{31 230., 31 698.}	20.0523	0.0972029
	newly 56 weeks cumul	44 760.	34 483.7	{34 221., 34 776.}	22.9588	0.113286																		
13	newly 3 weeks spec	7637.	7437.95	{7344., 7540.}	2.60875	0.0115037	newly 3 weeks cumul	33 066.	31 963.1	{31 808., 32 135.}	3.33536	0.0147359	newly 5 weeks spec	3594.	3325.63	{3246., 3391.}	7.46717	0.0337669	newly 5 weeks cumul	41 882.	40 318.1	{40 153., 40 488.}	3.73397	0.0165294
	newly 57 weeks cumul	49 021.	46 401.9	{46 230., 46 603.}	5.34291	0.0238492																		
14	newly 3 weeks spec	8364.	7086.12	{6940., 7188.}	15.2783	0.0720461	newly 3 weeks cumul	30 226.	25 113.6	{24 912., 25 296.}	16.914	0.08048	newly 5 weeks spec	4850.	4475.77	{4387., 4561.}	7.71608	0.0349253	newly 5 weeks cumul	41 750.	35 299.6	{35 062., 35 534.}	15.4501	0.0728926
	newly 50 weeks cumul	54 343.	49 036.2	{48 792., 49 256.}	9.76532	0.0446296																		
15	newly 3 weeks spec	7352.	6420.64	{6312., 6499.}	12.6681	0.0588562	newly 3 weeks cumul	31 019.	23 987.2	{23 808., 24 148.}	22.6694	0.111656	newly 5 weeks spec	3380.	3842.8	{3760., 3909.}	13.6923	0.0556822	newly 5 weeks cumul	39 368.	32 853.2	{32 640., 33 041.}	16.5486	0.0785707
	newly 51 weeks cumul	45 493.	44 156.4	{43 987., 44 340.}	2.93806	0.0129534																		
16	newly 3 weeks spec	8547.	6812.72	{6711., 6926.}	20.2911	0.0985346	newly 3 weeks cumul	32 656.	27 360.9	{27 109., 27 633.}	16.2149	0.0768436	newly 5 weeks spec	4015.	3259.77	{3183., 3341.}	18.8102	0.0905772	newly 5 weeks cumul	42 788.	35 398.5	{35 131., 35 644.}	17.27	0.0823435
	newly 54 weeks cumul	49 793.	41 542.2	{41 263., 41 837.}	16.5702	0.0786846																		
17	newly 3 weeks spec	6343.	3906.12	{3470., 6198.}	38.5341	0.219701	newly 3 weeks cumul	39 322.	26 418.4	{24 893., 35 479.}	32.8153	0.175849	newly 5 weeks spec	1376.	805.86	{651., 1493.}	45.0494	0.266323	newly 5 weeks cumul	43 673.	29 031.4	{27 182., 40 230.}	33.5255	0.181219
	newly 60 weeks cumul	44 678.	29 681.	{27 723., 41 721.}	33.5668	0.181853																		
18	newly 3 weeks spec	6778.	6576.29	{6470., 6689.}	2.9851	0.0132001	newly 3 weeks cumul	35 236.	32 773.4	{32 587., 32 933.}	6.98885	0.0314694	newly 5 weeks spec	2583.	2306.75	{2238., 2377.}	10.6949	0.049232	newly 5 weeks cumul	42 014.	39 017.	{38 830., 39 198.}	7.13343	0.0321439
	newly 59 weeks cumul	45 747.	42 170.3	{41 957., 42 338.}	7.81852	0.0353589																		
19	newly 3 weeks spec	8875.	6796.31	{6693., 6904.}	23.4219	0.115931	newly 3 weeks cumul	35 227.	27 333.3	{27 073., 27 546.}	22.4081	0.110194	newly 5 weeks spec	4524.	3258.37	{3162., 3347.}	27.9759	0.142641	newly 5 weeks cumul	46 101.	35 363.4	{35 048., 35 664.}	23.2914	0.115165
	newly 54 weeks cumul	56 361.	41 540.5	{41 222., 41 827.}	26.2956	0.132514																		
20	newly 3 weeks spec	6233.	6289.19	{6186., 6378.}	1.20953	0.00520823	newly 3 weeks cumul	33 857.	31 655.6	{31 462., 31 874.}	6.50214	0.0292038	newly 5 weeks spec	2013.	2221.45	{2150., 2291.}	10.3552	0.0426615	newly 5 weeks cumul	39 540.	37 660.2	{37 428., 37 866.}	4.75412	0.0211582
	newly 59 weeks cumul	42 220.	40 718.2	{40 470., 40 911.}	3.55699	0.015733																		
21	newly 3 weeks spec	6457.	5031.81	{4932., 5116.}	22.072	0.108351	newly 3 weeks cumul	36 224.	29 617.8	{29 421., 29 802.}	18.237	0.0874484	newly 5 weeks spec	1848.	1395.03	{1329., 1448.}	24.5114	0.122356	newly 5 weeks cumul	41 535.	33 683.3	{33 442., 33 883.}	18.9039	0.091005
	newly 60 weeks cumul	43 675.	35 170.5	{34 915., 35 389.}	19.4723	0.0940598																		
22	newly 3 weeks spec	7395.	5527.16	{5406., 5628.}	25.2581	0.126488	newly 3 weeks cumul	32 127.	25 838.8	{25 610., 26 066.}	19.5728	0.0946079	newly 5 weeks spec	2882.	2103.1	{2014., 2170.}	27.0264	0.137006	newly 5 weeks cumul	39 687.	31 391.2	{31 131., 31 648.}	20.9031	0.101851
	newly 56 weeks cumul	44 378.	34 454.7	{34 132., 34 759.}	22.3609	0.10993																		
23	newly 3 weeks spec	4881.	4506.9	{4404., 4596.}	7.66441	0.0346967	newly 3 weeks cumul	26 601.	23 379.1	{23 167., 23 600.}	12.112	0.0560831	newly 5 weeks spec	1370.	1503.77	{1441., 1557.}	9.77153	0.0402848	newly 5 weeks cumul	30 613.	27 517.1	{27 263., 27 730.}	10.113	0.0463129
	newly 57 weeks cumul	31 913.	29 487.	{29 261., 29 721.}	7.60207	0.0343465																		
24	newly 3 weeks spec	7357.	7085.93	{6992., 7188.}	3.68452	0.0163291	newly 3 weeks cumul	40 276.	34 414.7	{34 197., 34 634.}	14.5528	0.0683072	newly 5 weeks spec	2182.	2565.38	{2489., 2636.}	17.5701	0.0701921	newly 5 weeks cumul	46 540.	41 287.1	{41 075., 41 489.}	11.2869	0.0520155
	newly 59 weeks cumul	48 960.	44 868.3	{44 645., 45 083.}	8.35717	0.0379047																		
25	newly 3 weeks spec	9005.	7863.85	{7731., 7998.}	12.6724	0.0588868	newly 3 weeks cumul	36 976.	31 009.	{30 744., 31 239.}	16.1375	0.076439	newly 5 weeks spec	3489.	3894.26	{3807., 3977.}	11.6154	0.0476632	newly 5 weeks cumul	46 402.	40 539.8	{40 277., 40 779.}	12.6336	0.0586602
	newly 55 weeks cumul	51 783.	48 397.3	{48 160., 48 625.}	6.53832	0.0293697																		
26	newly 3 weeks spec	6466.	6908.49	{6804., 6995.}	6.84333	0.0287172	newly 3 weeks cumul	27 532.	26 660.9	{26 475., 26 873.}	3.16385	0.0139694	newly 5 weeks spec	2648.	3784.88	{3696., 3876.}	42.9335	0.155066	newly 5 weeks cumul	34 415.	35 610.6	{35 362., 35 802.}	3.47415	0.0148272
	newly 53 weeks cumul	38 357.	44 965.7	{44 768., 45 150.}	17.2296	0.0690344																		
27	newly 3 weeks spec	7481.	6921.59	{6818., 7029.}	7.47774	0.0337841	newly 3 weeks cumul	29 075.	26 684.7	{26 505., 26 832.}	8.22115	0.0372634	newly 5 weeks spec	4125.	3786.97	{3709., 3848.}	8.19467	0.0371898	newly 5 weeks cumul	38 835.	35 654.3	{35 448., 35 851.}	8.19024	0.0371154
	newly 53 weeks cumul	49 190.	44 999.7	{44 763., 45 197.}	8.51868	0.0386707																		
28	newly 3 weeks spec	5303.	3581.67	{3472., 3694.}	32.4596	0.170582	newly 3 weeks cumul	34 205.	25 158.9	{24 817., 25 495.}	26.4469	0.133424	newly 5 weeks spec	965.	696.19	{642., 745.}	27.856	0.142513	newly 5 weeks cumul	37 460.	27 456.5	{27 073., 27 785.}	26.7045	0.134945
	newly 60 weeks cumul	38 276.	27 988.7	{27 634., 28 309.}	26.8767	0.135965																		
29	newly 3 weeks spec	9186.	6517.92	{6389., 6636.}	29.0451	0.149061	newly 3 weeks cumul	36 850.	30 119.3	{29 901., 30 344.}	18.2652	0.0876002	newly 5 weeks spec	4278.	2455.02	{2381., 2542.}	42.6129	0.241329	newly 5 weeks cumul	47 405.	36 640.7	{36 362., 36 902.}	22.7071	0.111867
	newly 57 weeks cumul	55 913.	40 140.1	{39 848., 40 429.}	28.2097	0.14394																		
30	newly 3 weeks spec	9408.	6342.61	{6200., 6488.}	32.5828	0.171289	newly 3 weeks cumul	43 928.	35 722.2	{35 463., 35 954.}	18.6801	0.0898109	newly 5 weeks spec	3519.	1603.45	{1533., 1673.}	54.4345	0.341602	newly 5 weeks cumul	53 150.	40 563.	{40 273., 40 867.}	23.682	0.11738
	newly 60 weeks cumul	58 223.	42 062.9	{41 778., 42 353.}	27.7556	0.141202																		
31	newly 3 weeks spec	6334.	5951.99	{5676., 6507.}	7.0371	0.0318474	newly 3 weeks cumul	24 601.	22 414.6	{21 862., 23 659.}	8.88761	0.0406266	newly 5 weeks spec	3288.	3454.54	{3196., 4078.}	6.3163	0.0252251	newly 5 weeks cumul	32 650.	30 461.	{29 547., 33 179.}	7.20946	0.0326998
	newly 50 weeks cumul	39 652.	39 858.4	{38 026., 46 759.}	5.86054	0.0247213																		
32	newly 3 weeks spec	8242.	7189.54	{7067., 7291.}	12.7695	0.0593609	newly 3 weeks cumul	32 101.	29 213.1	{29 025., 29 371.}	8.99642	0.0409474	newly 5 weeks spec	4308.	3537.3	{3449., 3623.}	17.89	0.0856752	newly 5 weeks cumul	42 469.	37 863.	{37 674., 38 058.}	10.8455	0.0498604
	newly 56 weeks cumul	52 787.	45 232.4	{45 044., 45 420.}	14.3115	0.0670798																		
33	newly 3 weeks spec	5633.	2822.95	{2710., 2939.}	49.8855	0.300241	newly 3 weeks cumul	30 340.	21 358.3	{21 099., 21 606.}	29.6036	0.152473	newly 5 weeks spec	2012.	528.96	{478., 587.}	73.7097	0.581363	newly 5 weeks cumul	35 798.	23 116.9	{22 790., 23 393.}	35.4242	0.189954
	newly 60 weeks cumul	38 479.	23 506.3	{23 181., 23 777.}	38.9113	0.214063																		
34	newly 3 weeks spec	6383.	6074.66	{5973., 6181.}	4.83064	0.0215379	newly 3 weeks cumul	29 792.	26 009.2	{25 792., 26 184.}	12.6975	0.0589802	newly 5 weeks spec	2446.	2820.04	{2747., 2888.}	15.2919	0.0617191	newly 5 weeks cumul	36 225.	33 005.7	{32 823., 33 193.}	8.88698	0.040424
	newly 56 weeks cumul	39 694.	38 481.5	{38 247., 38 697.}	3.05454	0.0134766																		
35	newly 3 weeks spec	5611.	5016.99	{4917., 5102.}	10.5865	0.048645	newly 3 weeks cumul	36 155.	29 579.9	{29 362., 29 756.}	18.1858	0.087177	newly 5 weeks spec	1151.	1393.74	{1344., 1441.}	21.0895	0.0829203	newly 5 weeks cumul	39 920.	33 644.9	{33 455., 33 840.}	15.7192	0.0742757
	newly 60 weeks cumul	40 865.	35 133.	{34 923., 35 311.}	14.0267	0.0656409																		
36	newly 3 weeks spec	9648.	7529.01	{7413., 7651.}	21.963	0.10774	newly 3 weeks cumul	37 219.	31 402.4	{31 166., 31 633.}	15.628	0.0738089	newly 5 weeks spec	5029.	3235.8	{3144., 3309.}	35.6572	0.191593	newly 5 weeks cumul	49 093.	39 683.3	{39 345., 39 960.}	19.1672	0.0924187
	newly 56 weeks cumul	60 543.	44 989.7	{44 719., 45 318.}	25.6898	0.128956																		
37	newly 3 weeks spec	8031.	7408.61	{7287., 7565.}	7.74984	0.0352408	newly 3 weeks cumul	39 903.	31 885.9	{31 699., 32 182.}	20.0915	0.0974455	newly 5 weeks spec	2840.	3294.27	{3245., 3420.}	17.0482	0.0685963	newly 5 weeks cumul	47 625.	40 178.2	{40 059., 40 532.}	15.6364	0.0739287
	newly 57 weeks cumul	51 821.	46 163.8	{46 142., 46 614.}	10.9168	0.0503805																		
38	newly 3 weeks spec	6443.	6344.66	{6238., 6466.}	1.71845	0.00755492	newly 3 weeks cumul	29 746.	26 104.4	{25 900., 26 309.}	12.2422	0.056722	newly 5 weeks spec	2643.	3084.79	{2991., 3176.}	16.7155	0.0670314	newly 5 weeks cumul	36 633.	33 665.	{33 423., 33 892.}	8.10207	0.0367005
	newly 54 weeks cumul	40 381.	39 978.6	{39 758., 40 207.}	1.00545	0.00439267																		
39	newly 3 weeks spec	8056.	5519.97	{5394., 5640.}	31.48	0.16424	newly 3 weeks cumul	34 505.	25 821.9	{25 592., 26 016.}	25.1648	0.125907	newly 5 weeks spec	3317.	2096.72	{2027., 2164.}	36.7887	0.199347	newly 5 weeks cumul	43 007.	31 374.8	{31 126., 31 619.}	27.0472	0.136968
	newly 56 weeks cumul	48 356.	34 427.7	{34 158., 34 689.}	28.8037	0.147552																		
40	newly 3 weeks spec	7422.	6790.8	{6688., 6901.}	8.50445	0.0386324	newly 3 weeks cumul	30 770.	28 826.5	{28 611., 29 036.}	6.31635	0.028343	newly 5 weeks spec	3674.	3180.8	{3116., 3241.}	13.4241	0.0626684	newly 5 weeks cumul	39 721.	36 693.3	{36 493., 36 899.}	7.62239	0.0344372
	newly 57 weeks cumul	48 338.	43 010.9	{42 796., 43 213.}	11.0205	0.0507129																		
41	newly 3 weeks spec	7711.	6353.09	{6210., 6481.}	17.61	0.0841876	newly 3 weeks cumul	45 986.	35 735.6	{35 390., 36 107.}	22.2903	0.109537	newly 5 weeks spec	1635.	1599.37	{1532., 1667.}	3.21284	0.0142007	newly 5 weeks cumul	51 212.	40 568.6	{40 232., 40 919.}	20.7831	0.101192
	newly 60 weeks cumul	52 636.	42 067.5	{41 692., 42 353.}	20.0785	0.0973456																		
42	newly 3 weeks spec	7808.	5630.46	{5507., 5711.}	27.8886	0.14221	newly 3 weeks cumul	27 957.	21 992.2	{21 732., 22 169.}	21.3358	0.10431	newly 5 weeks spec	4650.	2955.57	{2839., 3040.}	36.4394	0.197463	newly 5 weeks cumul	38 862.	29 087.6	{28 691., 29 345.}	25.1515	0.125957
	newly 51 weeks cumul	51 353.	35 696.2	{35 133., 35 915.}	30.4886	0.158254																		
43	newly 3 weeks spec	8961.	8354.08	{8254., 8488.}	6.7729	0.0305063	newly 3 weeks cumul	34 781.	33 153.3	{32 955., 33 385.}	4.67991	0.0208277	newly 5 weeks spec	4598.	4133.35	{4041., 4217.}	10.1055	0.0464166	newly 5 weeks cumul	46 123.	43 246.4	{43 069., 43 510.}	6.23676	0.027985
	newly 56 weeks cumul	56 656.	51 713.8	{51 523., 51 990.}	8.72317	0.0396785																		
44	newly 3 weeks spec	7188.	5542.82	{5413., 5674.}	22.8879	0.112944	newly 3 weeks cumul	31 836.	25 857.7	{25 569., 26 134.}	18.7783	0.090342	newly 5 weeks spec	3246.	2106.77	{2033., 2164.}	35.0964	0.187863	newly 5 weeks cumul	39 959.	31 421.6	{31 098., 31 694.}	21.3653	0.104397
	newly 56 weeks cumul	46 072.	34 488.2	{34 195., 34 729.}	25.1428	0.125776																		
45	newly 3 weeks spec	7528.	5868.36	{5743., 5972.}	22.0462	0.108216	newly 3 weeks cumul	33 385.	29 197.1	{28 944., 29 432.}	12.5442	0.0582211	newly 5 weeks spec	3187.	2056.5	{1979., 2136.}	35.4722	0.190425	newly 5 weeks cumul	41 374.	34 760.9	{34 496., 35 049.}	15.9836	0.0756436
	newly 58 weeks cumul	46 827.	37 545.4	{37 271., 37 786.}	19.821	0.0959452																		
46	newly 3 weeks spec	8312.	3585.29	{3476., 3710.}	56.8661	0.365316	newly 3 weeks cumul	39 284.	25 157.1	{24 813., 25 505.}	35.961	0.193581	newly 5 weeks spec	3124.	702.18	{645., 756.}	77.523	0.649158	newly 5 weeks cumul	47 611.	27 473.1	{27 089., 27 816.}	42.2967	0.238821
	newly 60 weeks cumul	52 255.	28 005.3	{27 621., 28 335.}	46.4065	0.270907																		
47	newly 3 weeks spec	8223.	7869.06	{7724., 7990.}	4.30427	0.0191415	newly 3 weeks cumul	34 349.	31 012.	{30 820., 31 236.}	9.71487	0.0443897	newly 5 weeks spec	3657.	3888.36	{3794., 3968.}	6.3265	0.026566	newly 5 weeks cumul	43 642.	40 529.7	{40 275., 40 782.}	7.13143	0.0321361
	newly 55 weeks cumul	49 521.	48 402.8	{48 104., 48 634.}	2.25795	0.00992228																		
48	newly 3 weeks spec	8405.	7374.78	{7247., 7485.}	12.2572	0.0568447	newly 3 weeks cumul	39 417.	33 813.	{33 578., 34 007.}	14.2172	0.0666118	newly 5 weeks spec	2763.	2856.04	{2772., 2936.}	3.43829	0.0145426	newly 5 weeks cumul	47 174.	41 321.7	{41 043., 41 475.}	12.4058	0.0575398
	newly 58 weeks cumul	50 734.	45 600.2	{45 302., 45 742.}	10.1191	0.046353																		
49	newly 3 weeks spec	6054.	6064.26	{5967., 6151.}	1.00793	0.00437251	newly 3 weeks cumul	27 886.	25 978.5	{25 798., 26 150.}	6.84046	0.0307785	newly 5 weeks spec	2454.	2814.06	{2734., 2880.}	14.6724	0.0593791	newly 5 weeks cumul	34 341.	32 968.6	{32 787., 33 153.}	3.9963	0.0177164
	newly 56 weeks cumul	38 035.	38 459.2	{38 238., 38 663.}	1.11973	0.00483186																		
50	newly 3 weeks spec	8264.	7208.62	{7106., 7308.}	12.7708	0.0593662	newly 3 weeks cumul	33 965.	30 327.3	{30 132., 30 489.}	10.7102	0.0492031	newly 5 weeks spec	3752.	3302.23	{3207., 3387.}	11.9875	0.055548	newly 5 weeks cumul	43 375.	38 575.8	{38 389., 38 765.}	11.0646	0.0509282
	newly 57 weeks cumul	50 179.	44 793.2	{44 581., 45 003.}	10.7332	0.0493125																		
